# Development of an Antimicrobial Coating Film for Denture Lining Materials

**DOI:** 10.3390/pharmaceutics17070902

**Published:** 2025-07-11

**Authors:** Kumiko Yoshihara, Takeru Kameyama, Noriyuki Nagaoka, Yukinori Maruo, Yasuhiro Yoshida, Bart Van Meerbeek, Takumi Okihara

**Affiliations:** 1 National Institute of Advanced Industrial Science and Technology (AIST), Health and Medical Research Institute, Takamatsu 761-0395, Japan; 2 Department of Pathology & Experimental Medicine, Graduate School of Medicine, Dentistry and Pharmaceutical Sciences, Okayama University, Okayama 700-8558, Japan; 3 Graduate School of Natural Science and Technology, Okayama University, Okayama 700-8558, Japan; t.kameyama@hagihara.co.jp (T.K.); okihara@cc.okayama-u.ac.jp (T.O.); 4 Dental School, Advanced Research Center for Oral and Craniofacial Science, Okayama University, Okayama 700-8558, Japan; nagaoka@okayama-u.ac.jp; 5 Department of Prosthodontics, Okayama University, Okayama 700-8558, Japan; ykmar@md.okayama-u.ac.jp; 6 Department of Biomaterials and Bioengineering, Faculty of Dental Medicine, Hokkaido University, Sapporo 060-8586, Japan; yasuhiro@den.hokudai.ac.jp; 7 BIOMAT, Department of Oral Health Sciences, KU Leuvem, 3000 Leuven, Belgium; bart.vanmeerbeek@kuleuven.be

**Keywords:** antimicrobial, denture liner, cetylpyridinium chloride, drug release, drug recharge

## Abstract

**Background/Objectives:** Denture hygiene is essential for the prevention of oral candidiasis, a condition frequently associated with Candida albicans colonization on denture surfaces. Cetylpyridinium chloride (CPC)-loaded montmorillonite (CPC-Mont) has demonstrated antimicrobial efficacy in tissue conditioners and demonstrates potential for use in antimicrobial coatings. In this study, we aimed to develop and characterize CPC-Mont-containing coating films for dentures, focusing on their physicochemical behaviors and antifungal efficacies. **Methods:** CPC was intercalated into sodium-type montmorillonite to prepare CPC-Mont; thereafter, films containing CPC-Mont were fabricated using emulsions of different polymer types (nonionic, cationic, and anionic). CPC loading, release, and recharging behaviors were assessed at various temperatures, and activation energies were calculated using Arrhenius plots. Antimicrobial efficacy against Candida albicans was evaluated for each film using standard microbial assays. **Results:** X-ray diffraction analysis confirmed the expansion of montmorillonite interlayer spacing by approximately 3 nm upon CPC loading. CPC-Mont showed temperature-dependent release and recharging behavior, with higher temperatures enhancing its performance. The activation energy for CPC release was 38 kJ/mol, while that for recharging was 26 kJ/mol. Nonionic emulsions supported uniform CPC-Mont dispersion and successful film formation, while cationic and anionic emulsions did not. CPC-Mont-containing coatings maintained antimicrobial activity against Candida albicans on dentures. **Conclusions:** CPC-Mont can be effectively incorporated into nonionic emulsion-based films to create antimicrobial coatings for denture applications. The films exhibited temperature-responsive, reversible CPC release and recharging behaviors, while maintaining antifungal efficacy, findings which suggest the potential utility of CPC-Mont-containing films as a practical strategy to prevent denture-related candidiasis.

## 1. Introduction

Many elderly individuals continue to use dentures, and prolonged use can promote the growth of oral bacteria and fungi, increasing the risk of infections such as denture stomatitis [1]. In particular, Candida species tend to adhere to the denture base, contributing to persistent inflammation and discomfort [2,3]. The porous nature of the denture surface facilitates bacterial and plaque adhesion, making proper cleaning difficult; in particular, indirect relining, which involves curing the denture material intraorally, often results in bubble entrapment and surface roughness, creating a favorable environment for plaque accumulation [3,4]. Furthermore, elderly and care-dependent individuals may face challenges in maintaining proper denture hygiene, leading to more pronounced bacterial biofilm accumulation [4].

To address this issue, we propose applying an antimicrobial coating to dentures to inhibit microbial adhesion and biofilm formation on the denture surface. Additionally, such coatings could simplify denture cleaning for elderly individuals with impaired vision or dexterity, thereby improving their quality of life (QOL).

In recent years, antimicrobial coatings utilizing antimicrobial polymers, silver, and metal oxides have been developed, all showing promise in food and clinical applications [5]. However, for an antimicrobial coating to be effectively used in the oral cavity, it must exhibit high biocompatibility and maintain antimicrobial efficacy over an extended period [6]. In this study, we considered using cetylpyridinium chloride-loaded montmorillonite (CPC-Mont), which has already been approved as a tissue conditioner. Montmorillonite is a clay mineral with cation exchange capacity, which allows for the formation of intercalated complexes with polymers and various organic compounds [7]. By leveraging this property, it is possible to develop a CPC carrier that exhibits both sustained release and recharging capabilities.

The development of antimicrobial polymer materials typically involves one of the two following approaches: incorporating antimicrobial agents or carrier particles into the base material, or attaching them to the surface [8]. However, incorporating solid particles into the base material may compromise its physical properties, while surface attachment can lead to reduced antimicrobial longevity due to depletion or washout of the antimicrobial agent [9,10]. Additionally, excessive release of high concentrations of antimicrobial agents over a short period poses potential risks to biological tissues. Therefore, it is essential to develop antimicrobial materials that maintain strong and long-lasting antimicrobial effects without compromising the physical properties of the base material. In this study, we explored the use of CPC-Mont, dispersed in an emulsion-based film, for denture coating.

In this study, we investigated the dispersion behavior of an intercalated compound composed of montmorillonite and the cationic surfactant cetylpyridinium chloride (CPC) in an emulsion system; furthermore, we applied the dispersed material onto a polyester substrate and evaluated its drug release behavior using absorbance measurements, analyzing how the emulsion affects the CPC release kinetics and recharging properties of the montmorillonite intercalated complex. Additionally, we coated denture materials with this formulation and assessed its antimicrobial activity against Candida albicans.

## 2. Materials and Methods

### 2.1. Synthesis of CPC-Mont

A cetylpyridinium chloride (CPC, Sigma-Aldrich, St. Louis, MO, USA) solution was prepared by mixing CPC with ultrapure water to obtain a final concentration of 200 ppm. Subsequently, 0.0115 g of montmorillonite (Kunipia-F, Kunimine Industries Co., Ltd., Tokyo, Japan) was immersed in 15 mL of the CPC solution and left to stand. At predetermined time intervals, the supernatant was collected, and its absorbance was measured using a spectrophotometer (UV-1800, Hitachi, Tokyo, Japan) to determine the decrease in CPC concentration. The amount of CPC adsorbed onto the montmorillonite was calculated by subtracting the maximum decrease in CPC concentration from the initial concentration, dividing the difference by the amount of montmorillonite, and then converting the result to moles per gram of montmorillonite.

### 2.2. X-Ray Diffraction (XRD) Analysis of CPC–Montmorillonite

The synthesized CPC–montmorillonite and montmorillonite were analyzed using an X-ray diffractometer (Smart Lab, Rigaku, Tokyo, Japan) to determine their interlayer spacing. The interlayer distance for each was then calculated using Bragg’s equation.

### 2.3. Evaluation of the Dispersibility of CPC–Montmorillonite Intercalation Compounds in Emulsions

The following five types of emulsions were used: three acrylic component emulsions (AE-850 (Resonac Holdings Corporation, Tokyo, Japan), AE-803 (Resonac Holdings Corporation), and F-375 (Resonac Holdings Corporation)) and two polyester emulsions, KT-0507 (Unitika LTD., Osaka, Japan), and KT-8701 (Unitika LTD.) (Table 1). Each emulsion was diluted 100 times with distilled water, followed by the addition of 0.1 g of CPC–montmorillonite intercalation compound. The mixture was manually shaken, and its dispersibility was evaluated.

### 2.4. Evaluation of the Sustained Release Behavior of CPC–Montmorillonite and the CPC Loading Behavior of Montmorillonite

To evaluate sustained release behavior, 0.03 g of CPC–montmorillonite was immersed in ultrapure water and kept under static conditions at 6 °C, 17 °C, 27 °C, and 40 °C. At predetermined time intervals, the supernatant was collected, and its absorbance was measured. The results were plotted using the Arrhenius equation to calculate the activation energy.

Additionally, 0.015 g of montmorillonite was immersed in 5 mL of a CPC aqueous solution with an initial concentration of 200 ppm. The mixture was maintained at 6 °C, 17 °C, 27 °C, and 40 °C under static conditions. At predetermined time intervals, the supernatant was collected, and its absorbance was measured to assess the CPC loading behavior.

### 2.5. Calculation of Activation Energy

The release amount of CPC was measured at 6, 17, 27, and 40 °C, and the reaction rate constant (*k*) was determined. The activation energy (*Ea*) was calculated using the Arrhenius equation, as follows:

*k* = *Ae* − *Ea*/*RT*

where *k* is the reaction rate constant, *A* is the pre-exponential factor, *Ea* is the activation energy (J/mol), *R* is the universal gas constant (8.314 J/mol·K), and *T* is the absolute temperature (K). [11].

### 2.6. Evaluation of the Sustained Release Behavior of CPC–Montmorillonite Dispersed in a Polymer

The CPC–montmorillonite intercalation compound and the emulsion AE-850, which exhibited good dispersibility, were used for this experiment. A mixture of ultrapure water, emulsion, and CPC–montmorillonite was prepared at a ratio of 1 mL:0.08 mL:0.04 g and dispersed using a Violamo homogenizer (VH-10, As One, Osaka, Japan). The resulting dispersion was then used to create a film with a thickness of 200 µm by employing a film applicator (BEVS, Huangpu District, Guangzhou, China), ensuring the dispersion of CPC–montmorillonite within the polymer.

The prepared film was evaluated for its sustained release behavior using the same absorbance measurement method as applied to the CPC-Mont powder in Section 2.4.

### 2.7. Evaluation of the CPC Loading Behavior in Films Containing Montmorillonite or CPC–Montmorillonite

A mixture of ultrapure water, emulsion, and montmorillonite was prepared at a ratio of 1 mL:0.08 mL:0.019 g, and a polymer film with dispersed montmorillonite was fabricated using the method described above; this film was then immersed in a CPC aqueous solution with an initial concentration of 200 ppm, and the CPC loading behavior was evaluated using the same method.

Additionally, the CPC–montmorillonite dispersion film was first immersed in water to release CPC. After CPC release, the film was immersed in a CPC aqueous solution with an initial concentration of 200 ppm, and its reloading behavior was evaluated using the same method. Similarly, the film containing montmorillonite dispersed in the polymer was also immersed in the CPC aqueous solution (200 ppm), and its CPC loading behavior was assessed using the same approach.

### 2.8. Evaluation of the CPC Re-Release Behavior from Films Containing Dispersed Montmorillonite and CPC–Montmorillonite

The montmorillonite or CPC–montmorillonite films prepared above were immersed in ultrapure water to release CPC. The CPC re-release behavior was then evaluated using the same method as applied to the powdered samples.

### 2.9. Antimicrobial Test

#### 2.9.1. Fabrication of Denture Disks and Surface Treatments

Denture liner disks (Tokuyama Rebase 3, Tokuyama Dental, Tokyo, Japan) with dimensions of 10 × 2 mm were fabricated using silicone molds. All disks were standardized in size and surface finish. The surface treatments applied to the disks included CPC–montmorillonite (CPC-Mont) films, montmorillonite (Mont) films, CPC-released CPC-Mont films, CPC-charged Mont films, and CPC-recharged CPC-Mont films. Three disks were prepared for each treatment group. Untreated denture disks were used as controls.

#### 2.9.2. Cell Preparation

Candida albicans (ATCC 48130) was cultured in yeast mold (YM) broth (Becton, Dickinson and Company, Franklin Lakes, NJ, USA) under aerobic conditions at 37 °C for 24–48 h, ensuring that the cells were in the mid-logarithmic growth phase. The optical density (OD) at 600 nm was adjusted to 0.1 using a UV spectrophotometer (BioSpectrometer Basic, Eppendorf, Hamburg, Germany).

#### 2.9.3. Microbial Growth

Denture liner disks were placed in a 12-well polystyrene culture plate (Corning, NY, USA), where each disk was inoculated with 2 mL of C. albicans suspension (10^6^ cells/mL) in YM broth and incubated at 37 °C for 24 h. As controls, three wells were left without a material disk (positive control), and another three wells were left without the microbial suspension (negative control). The absorbance at 600 nm was measured over 24 h at 37 °C using a microplate reader (POLARstar Omega, BMG Labtech, Ortenberg, Germany). The experiment was performed in triplicate (*n* = 3 sites per disk, 3 disks per group).

#### 2.9.4. Statistical Analysis

Statistical analysis was conducted using one-way ANOVA, followed by Tukey’s honestly significant difference (HSD) test at a significance level of α = 0.05, using SPSS (Version 25, IBM, Armonk, NY, USA)

### 2.10. Cytotoxicity Test

The denture liner disks (10 mm diameter × 2 mm) were prepared, after which their surfaces were coated. All samples were immersed in 2 mL Dulbecco’s minimum essential medium (DMEM; Sigma-Aldrich) with 5% fetal bovine serum (FBS) and 1% penicillin–streptomycin for 24 h in a 37 °C incubator, upon which each solution was collected. Chinese hamster fibroblasts (V79-4, ATCC CCL-93) were routinely cultivated in Dulbecco’s minimum essential medium (DMEM) (Sigma-Aldrich, St Louis, MO, USA) with streptomycin at 37 °C and under 5% CO2. In the medium, 100 mL cells were seeded in 96-well plates, at 5 × 103 cells per well, and incubated for 24 h at 37 °C. The medium was next replaced with medium containing either undiluted sample extract or 50% or 10% diluted sample extract. For each group, cells were seeded into 3 wells. After 24 h incubation at 37 °C, the medium was removed, upon which cell survival was determined using an MTT assay (Sigma-Aldrich). After adding 10 mL MTT solution to each well, the cells were incubated for an additional 4 h. The resulting formazan crystals were dissolved by replacing the culture medium in each well with 90 mL dimethyl sulfoxide (Sigma-Aldrich). After storing the plates overnight, the absorbance at 450 nm was determined using a microplate reader (Multiskan Ascent 96/384, Thermo Electron, Waltham, MA, USA). The above-described procedure was repeated three times. Statistical analysis was performed with two-way ANOVA and Scheffe tests at the α = 0.05 significance level using IBM SPSS Statistics.

## 3. Results

### 3.1. Evaluation of CPC Loading onto Montmorillonite

**Figure 1:**
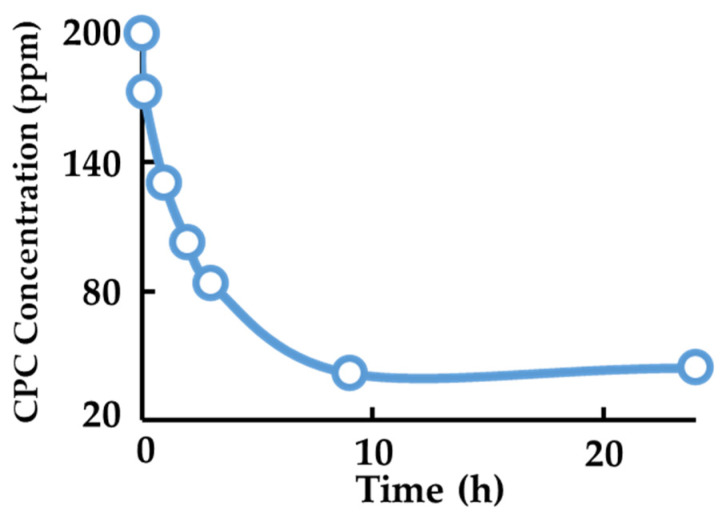
Evaluation of CPC loading onto montmorillonite.

The decrease in CPC concentration in the aqueous solution after the immersion of montmorillonite at room temperature is shown in Figure 1. The amount of CPC loaded onto montmorillonite increased over time, and the maximum loading capacity was reached after 9 h under room-temperature conditions (20 °C). Based on these results, it was determined that montmorillonite can load 5.8 × 10^−4^ mol of CPC per gram.

Montmorillonite (Mont) was added to a 200 ppm CPC solution, and the supernatant was collected at various time points. The absorbance of the supernatant was measured to evaluate the decrease in CPC concentration.

### 3.2. X-Ray Diffraction Analysis of CPC–Montmorillonite

**Figure 2:**
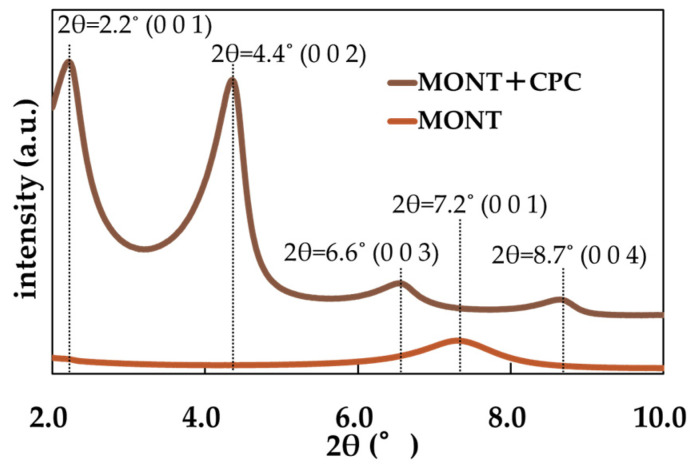
XRD analysis of CPC-Mont and montmorillonite (Mont).

X-ray diffraction (XRD) measurements were performed for both CPC–montmorillonite and pristine montmorillonite in the 2θ range of 2.0° to 10.0° (Figure 2).

In the 2.0° to 10.0° range, the characteristic peaks of the CPC–montmorillonite intercalation compound were observed at 2.2°, 4.4°, 6.6°, and 8.7°, while the peak for pristine montmorillonite appeared at 7.2°; these peaks were assigned to the (001) plane and analyzed using Bragg’s equation. Based on the calculations, the interlayer spacing was determined to be 1.24 nm for pristine montmorillonite and 4.0 nm for CPC–montmorillonite.

Since the thickness of a single montmorillonite layer is known to be 0.96 nm, the interlayer distance was calculated as 0.28 nm for pristine montmorillonite and 3.0 nm for CPC–montmorillonite. The expansion of the interlayer spacing in CPC–montmorillonite is attributed to the intercalation of CPC, a cationic surfactant with a larger molecular size than the original exchangeable cations present in the montmorillonite layers.

### 3.3. Evaluation of the Dispersion of CPC-Mont Intercalation Compounds in Emulsions

**Figure 3:**
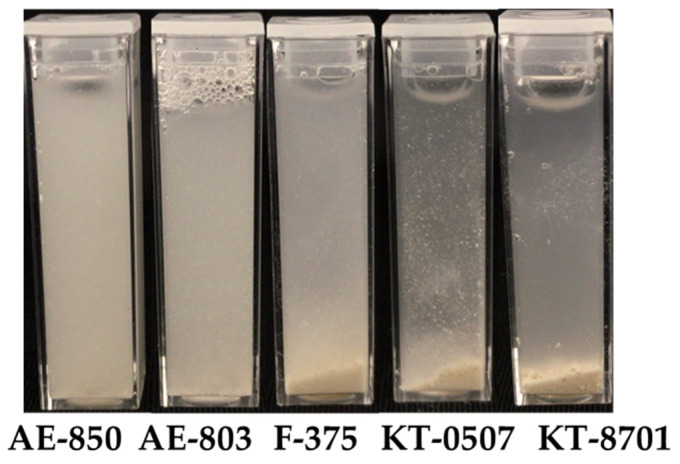
Evaluation of CPC-Mont dispersion in diluted emulsions.

The dispersion of CPC-Mont intercalation compounds in five different emulsions, each diluted 100 times, is shown in Figure 3. CPC-Mont intercalation compounds were well dispersed in nonionic and cationic emulsions; in contrast, in anionic emulsions, the CPC-Mont intercalation compounds exhibited aggregation.

### 3.4. Evaluation of the Release Behavior of CPC-Mont Intercalation Compounds and the CPC Loading Behavior of Montmorillonite

**Figure 4:**
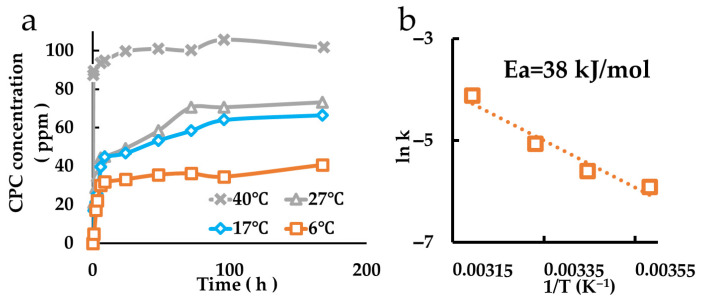
Measurement of CPC release amount. (**a**) Release behaviors of the samples at different temperatures. (**b**) Arrhenius plot.

The release behavior of CPC from CPC-Mont intercalation compounds is shown in Figure 4a. As the temperature of the ultrapure water in which the CPC-Mont intercalation compounds were immersed increased, the release duration shortened, indicating temperature-dependent release behavior. An Arrhenius plot (Figure 4b) revealed that the activation energy for CPC release from CPC-Mont intercalation compounds was 38 kJ/mol.

### 3.5. Evaluation of CPC Loading Behavior in Montmorillonite

**Figure 5:**
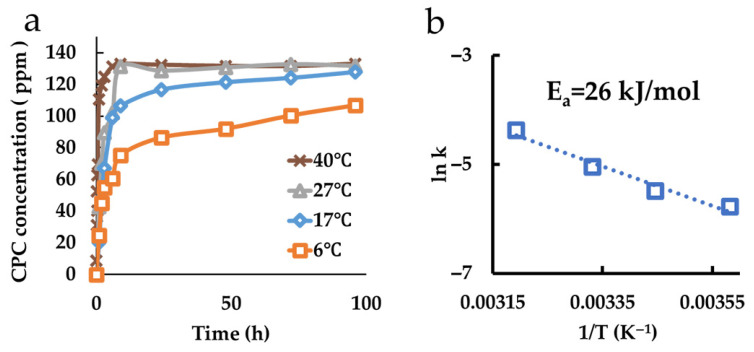
Measurement of CPC loading amount. (**a**) CPC loading behaviors at different temperatures, determined by subtracting the measured amount from the initial CPC concentration. (**b**) Arrhenius plot.

The CPC loading behavior in montmorillonite is shown in Figure 5a. In this graph, the vertical axis represents the concentration of CPC loaded onto montmorillonite, calculated by subtracting the measured amount from the initial concentration of 200 ppm. As the temperature of the CPC aqueous solution in which montmorillonite was immersed increased, the loading period shortened, indicating temperature-dependent behavior. An Arrhenius plot (Figure 5b) showed that the activation energy for CPC loading into montmorillonite was 26 kJ/mol.

### 3.6. Fabrication of CPC-Mont Dispersed Film

**Figure 6:**
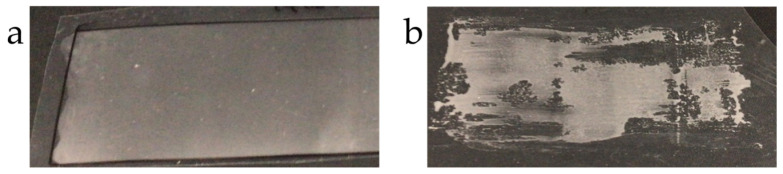
Photographs of film formation from CPC-Mont dispersed emulsions. (**a**) Films that were successfully fabricated. (**b**) Films that could not be fabricated.

Films could be successfully fabricated only with the nonionic acrylate ester emulsion (Figure 6a); in contrast, film fabrication was challenging with other emulsions (Figure 6b).

### 3.7. Evaluation of CPC Release Behavior from CPC-Mont Dispersed Emulsion Films

**Figure 7:**
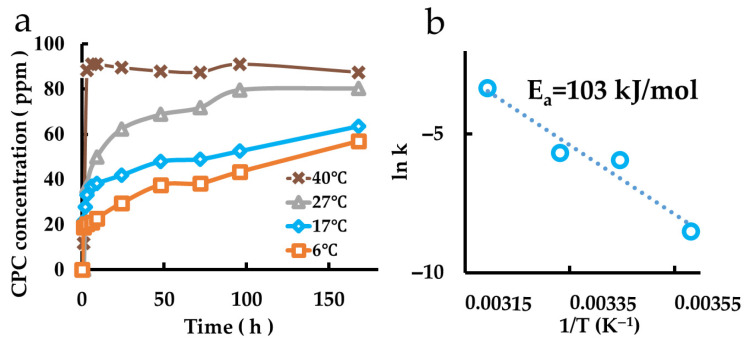
Measurement of CPC release from CPC-Mont dispersed in films. (**a**) Release behaviors of the samples at different temperatures. (**b**) Arrhenius plot.

The CPC release behaviors from films coated with CPC-Mont intercalated compounds dispersed in polymers are shown in Figure 7a. An Arrhenius plot (Figure 7b) revealed that the activation energy was 103 kJ/mol.

### 3.8. Evaluation of CPC Charge Behavior from Mont Dispersed Emulsion Films

**Figure 8:**
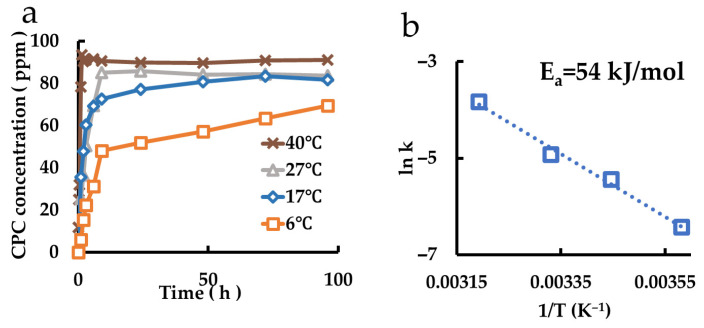
Measurement of CPC charge from Mont dispersed in films. (**a**) Charge behaviors of the samples at different temperatures. (**b**) Arrhenius plot.

The release behaviors of films coated with polymers in which montmorillonite was dispersed are shown in Figure 8a. Based on the results of an Arrhenius plot (Figure 8b), the activation energy was determined to be 54 kJ/mol.

### 3.9. Evaluation of CPC Charge Behavior from CPC Released CPC-Mont Dispersed Emulsion Films

**Figure 9:**
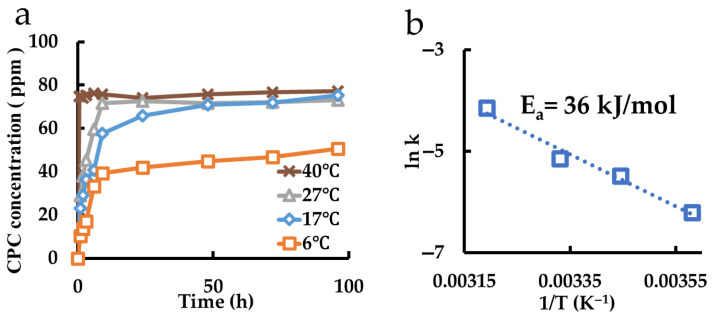
Measurement of CPC recharge from CPC released CPC-Mont dispersed in films. (**a**) Recharge behaviors of the samples at different temperatures. (**b**) Arrhenius plot.

The re-release behaviors of films coated with polymers in which CPC–montmorillonite intercalation compounds were dispersed are shown in Figure 9a. Based on the results of an Arrhenius plot (Figure 9b), the activation energy was determined to be 36 kJ/mol.

### 3.10. Evaluation of the CPC Release Behavior of CPC Charged Mont and CPC-Mont Dispersed Film

**Figure 10:**
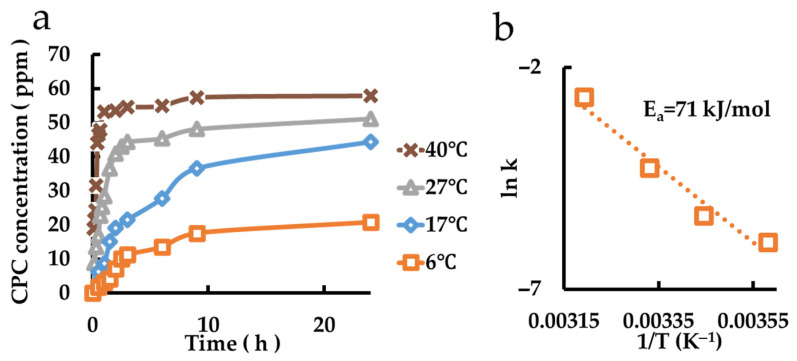
Measurement of CPC release from CPC charged Mont dispersed in films. (**a**) Release behaviors of the samples at different temperatures. (**b**) Arrhenius plot.

The sustained release behavior of the film coated with montmorillonite dispersed in polymer is shown in Figure 10a. According to the results of an Arrhenius plot (Figure 10b), the activation energy is 71 kJ/mol.

### 3.11. Evaluation of the CPC Re-Release Behavior of CPC Recharged CPC-Mont Dispersed Film

**Figure 11:**
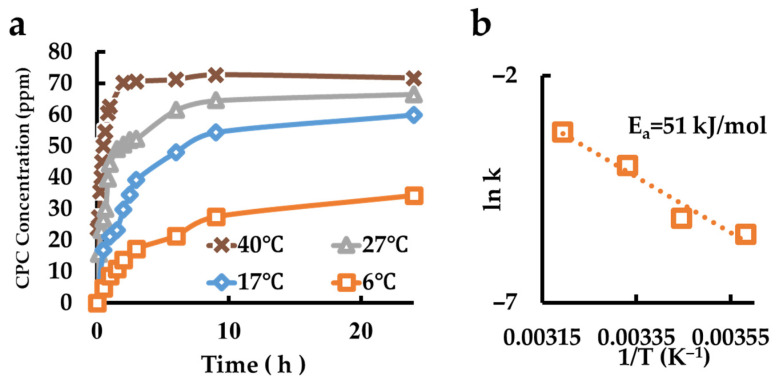
Measurement of CPC re-release from CPC recharged CPC-Mont dispersed in films. (**a**) Release behaviors of the samples at different temperatures. (**b**) Arrhenius plot.

The sustained release behavior of a film coated with a dispersion of CPC–montmorillonite interlayer compound in polymer is shown in Figure 11a. An Arrhenius plot resulted in an activation energy of 51 kJ/mol (Figure 11b).

### 3.12. Antimicrobial Test

**Figure 12:**
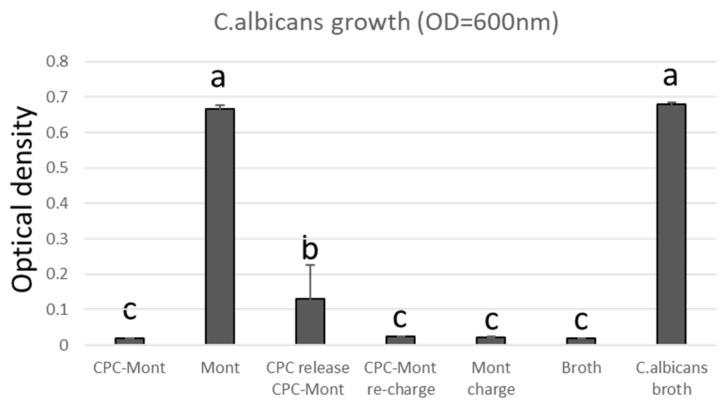
Antimicrobial test of Candida albicans on film-coated denture base material at 24 h. Error bars represent standard deviations. Different letters indicate statistically significant differences (*p* < 0.05). CPC-Mont-containing film demonstrated significant inhibition of Candida albicans growth.

The antimicrobial efficacy of CPC-Mont-containing films coated onto denture relining materials was evaluated by measuring the optical density of Candida albicans cultures (Figure 12). The denture relining material coated with a CPC-Mont-containing film exhibited low turbidity, indicating effective inhibition of C. albicans growth. In contrast, the Mont-containing film showed turbidity levels comparable to those of the uncoated denture relining material and the microbial suspension alone, demonstrating no significant antimicrobial effect. The CPC-Mont-containing film, from which CPC had been released, exhibited lower turbidity than the microbial suspension alone, but showed higher turbidity compared to the CPC-Mont-coated denture relining material. Furthermore, CPC-recharged CPC-Mont-containing and Mont-containing film-coated denture relining materials exhibited antimicrobial activity.

### 3.13. Cytotoxicity Test

**Figure 13:**
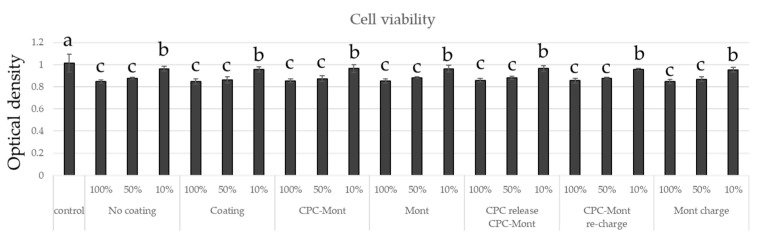
Cytotoxicity of the experimental resin liner with different coatings, as determined using an MTT assay. Error bars represent standard deviations. Different letters indicate statistically significant differences (*p* < 0.05). There was no difference in toxicity depending on the amount of CPC loaded.

The control with no sample had the least toxicity (Figure 13). All other controls, including those without coating, showed the same trend. Those without dilution showed slightly more toxicity, and toxicity decreased with increasing dilution. However, there was no difference in toxicity depending on the amount of CPC loaded.

## 4. Discussion

In this study, we investigated a coating film designed to confer antimicrobial properties to dentures, examining the application of CPC-loaded montmorillonite (CPC-Mont), an antimicrobial agent previously utilized in tissue conditioners. Our findings revealed that the film composition influenced the dispersibility of CPC-Mont, and that CPC release and charge required higher activation energy in CPC-Mont-containing films compared to CPC-Mont alone. Moreover, CPC-Mont-containing films were capable of charging CPC, similar to CPC-Mont- and Mont-containing films, and all exhibited antimicrobial activity against Candida albicans.

CPC-Mont has been explored as a rechargeable inorganic antimicrobial agent for dental materials. It has been incorporated into dental adhesives and tissue conditioners to evaluate its antimicrobial efficacy. Tissue conditioners consist of a powder phase, composed of methacrylate ester copolymers and polymethylmethacrylate, and a liquid phase, containing fatty acid esters and ethanol; these materials gel upon mixing and are applied to the denture’s inner surface. A CPC-Mont-containing tissue conditioner has been approved for medical use and is commercially available as Tissue Conditioner CPC [12].

Montmorillonite is a layered silicate compound composed of an octahedral Al-O sheet sandwiched between two tetrahedral Si-O sheets, forming a sandwich-like structure. The montmorillonite used in this study was sodium-type montmorillonite, which has a layered structure with sodium ions in its interlayer space, capable of accommodating various molecules. CPC-Mont was obtained by intercalating CPC into the montmorillonite interlayer. X-ray diffraction (XRD) analysis confirmed that CPC insertion expanded interlayer spacing by 3 nm, indicating that CPC, a cationic surfactant larger than sodium ions, had intercalated and increased the interlayer distance [7], a result which aligns with findings in the previous literature.

Cetylpyridinium chloride (CPC) has been widely used as an active ingredient in mouthwashes and oral rinses due to its antimicrobial properties [13,14,15]; it has been shown to effectively reduce oral bacteria and prevent plaque formation. CPC has also been incorporated into various oral care products, including throat sprays and lozenges, for its antimicrobial and antiviral effects; its long history of safe use in dental and medical applications highlights its importance in oral hygiene [15].

While previous studies have evaluated the loading capacity of CPC onto montmorillonite, its temperature dependency has not been reported. In our study, we measured CPC loading capacity and time at different temperatures (6 °C, 17 °C, 27 °C, and 40 °C), revealing that higher temperatures led to faster and greater CPC loading; the specific temperatures (6 °C, 17 °C, 27 °C, and 40 °C) were selected to enable calculation of the activation energy required for the sustained release of CPC using the Arrhenius equation, which relies on the linear relationship between the inverse of the absolute temperature and the logarithm of the release rate constant. When expressed in Kelvin, the selected temperatures correspond to 279 K, 290 K, 300 K, and 313 K, respectively. We intentionally did not include 37 °C (310 K) in order to ensure a wider temperature range, thereby improving the linearity and reliability of the Arrhenius plot. Additionally, CPC release from CPC-Mont was also enhanced at higher temperatures. Given that body temperature is approximately 36 °C, these findings suggest that CPC release may be efficient in vivo. Based on Arrhenius plots, we calculated the activation energy for CPC release and charge. The activation energy for CPC release was 38 kJ/mol, whereas that for charge was 26 kJ/mol. The lower activation energy for charge suggests that larger organic cations preferentially intercalate into montmorillonite over inorganic cations.

We also evaluated the suitability of film formulations for denture application. The dispersibility of CPC-Mont varied depending on the film composition. To investigate the effects of polymer types, we used nonionic, cationic, and anionic emulsions [16]. The dispersibility of CPC-Mont differed significantly depending on the polymer type. CPC-Mont was well dispersed in nonionic and cationic emulsions, but aggregated in anionic emulsions. In nonionic emulsions, no interactions occurred between the emulsion and CPC-Mont, allowing for uniform dispersion. In cationic emulsions, negatively charged ions in the aqueous phase interacted with the anionic surface of Mont and repelled CPC-Mont, promoting dispersion [17]; in contrast, in anionic emulsions, positively charged ions interacted with the emulsion interface, leading to the aggregation of CPC-Mont. Furthermore, while films were successfully formed using the nonionic emulsion, film formation failed with the cationic and anionic emulsions, likely due to non-uniform dispersion inhibiting uniform film formation. The above findings highlight the importance of selecting an appropriate base material for CPC-Mont-containing films.

The temperature dependence of the sustained release behavior of CPC from montmorillonite embedded in resin was determined, and the activation energy was obtained using the Arrhenius equation [18]. Although there is no report evaluating sustained release from montmorillonite itself, the value is similar to the activation energy obtained when it is ion-exchanged, as described in the literature [19]. It is reasonable to assume that if polymer chains are buried around montmorillonite, the activation energy would be higher because of the additional activation energy required to move the polymer chains, in addition to the activation energy required to extend the interlayer distance of montmorillonite.

Films containing CPC-Mont were prepared using the nonionic emulsion and analyzed. Similar to CPC-Mont alone, CPC release and charge from the films exhibited temperature dependence, with higher temperatures leading to faster and greater release and charge. The activation energy for CPC release was higher in films than in CPC-Mont alone, likely due to interactions between montmorillonite and the polymer chains, which hinder CPC release (Table 2). Additionally, the activation energy for the re-release was lower than that for the initial release, suggesting that repeated release and charge reduce the polymer chain-induced inhibition. Films containing montmorillonite with a wider initial interlayer spacing facilitated CPC release more effectively, exhibiting lower activation energy compared to films containing montmorillonite with a shorter interlayer distance.

The activation energy for CPC charge was lowest in montmorillonite alone, followed by montmorillonite-dispersed films, and highest in CPC-Mont-dispersed films after CPC release (Table 3). The increased activation energy in CPC-Mont-containing films after CPC release is attributed to the wider interlayer spacing, which reduces the energy required to overcome polymer chain resistance.

The antimicrobial test was conducted to target dentures using Candida albicans, a major causative agent of oral candidiasis [20], an opportunistic infection caused by commensal Candida species, which, if untreated, can spread from the oral mucosa to the pharynx, esophagus, lungs, and bloodstream [21,22]. Although Candida is a commensal microorganism, it can proliferate under conditions such as chemotherapy, steroid therapy, diabetes, AIDS, and systemic weakening, leading to opportunistic infections [23,24]. Since dentures promote Candida adhesion, inadequate denture hygiene increases the risk of oral candidiasis and contributes to denture maladaptation, making prevention essential [25,26].

CPC is a broad-spectrum antimicrobial agent effective against bacteria and fungi, including SARS-CoV-2 (COVID-19) [27]; it is widely used in oral care products, such as lozenges, toothpaste, and mouthwash, as well as in cosmetics and industrial applications. Previous studies on tissue conditioners demonstrated that 2% CPC-Mont exhibited antimicrobial activity against Candida albicans [12]. Similarly, the results of our study confirmed its antimicrobial efficacy. As the developed film forms a thin coating on dentures for short-term use (e.g., daily application), long-term antimicrobial performance was not evaluated. Additionally, optimizing the CPC concentration is necessary to prevent the mucosal irritation potentially caused by high CPC levels.

## 5. Conclusions

In this study, we investigated the fundamental properties of antimicrobial films for denture applications; the results demonstrated that CPC loading expanded the interlayer spacing of montmorillonite; CPC-Mont-containing films dispersed well in nonionic and cationic emulsions, with nonionic emulsions being more suitable for film formation; the presence of polymer chains influenced CPC charge and release behavior in montmorillonite; and the CPC-Mont-loaded film exhibited reversible CPC charge and release while maintaining antimicrobial activity against Candida albicans. The above findings suggest the potential of CPC-Mont-containing films for antimicrobial denture applications.

## Figures and Tables

**Table 1:** Specifications of the materials used in this study.

Sample Name	Company	Component	Charge
AE-850	Resonac Holdings Corporation, Tokyo, Japan	acrylic	nonionic
AE-803	Resonac Holdings Corporation, Tokyo, Japan	acrylic	cationic
F-375	Resonac Holdings Corporation, Tokyo, Japan	acrylic	anionic
KT-0507	Unitika LTD., Osaka, Japan	polyester	anionic
KT-8701	Unitika LTD., Osaka, Japan	polyester	anionic

**Table 2:** CPC Release Profile.

Material	Polymer	Ea (kJ/mol)
CPC-Mont	-	38
CPC-Mont Film	AE-850	103
CPC Charge Mont Film	AE-850	71
CPC Recharge CPC-Mont Film	AE-850	51

**Table 3:** CPC Loading Behavior.

Material	Polymer	Ea (kJ/mol)
CPC-Mont	-	26
Mont Film	AE-850	54
CPC Released CPC-Mont Film	AE-850	36

## Data Availability

The original contributions presented in this study are included in the article. Further inquiries can be directed to the corresponding author.

## References

[1] Stoopler E.T., Villa A., Bindakhil M., Díaz D.L.O., Sollecito T.P. (2024). Common Oral Conditions: A Review. JAMA.

[2] Le Bars P., Kouadio A.A., Amouriq Y., Bodic F., Blery P., Bandiaky O.N. (2024). Different Polymers for the Base of Removable Dentures? Part II: A Narrative Review of the Dynamics of Microbial Plaque Formation on Dentures. Polymers.

[3] McReynolds D.E., Moorthy A., Moneley J.O., Jabra-Rizk M.A., Sultan A.S. (2023). Denture stomatitis-An interdisciplinary clinical review. J. Prosthodont..

[4] Niesten D., Witter D.J., Bronkhorst E.M., Creugers N.H.J. (2017). Oral health care behavior and frailty-related factors in a care-dependent older population. J. Dent..

[5] Omerović N., Djisalov M., Živojević K., Mladenović M., Vunduk J., Milenković I., Knežević N.Ž., Gadjanski I., Vidić J. (2021). Antimicrobial nanoparticles and biodegradable polymer composites for active food packaging applications. Compr. Rev. Food Sci. Food Saf..

[6] Butler J., Handy R.D., Upton M., Besinis A. (2023). Review of Antimicrobial Nanocoatings in Medicine and Dentistry: Mechanisms of Action, Biocompatibility Performance, Safety, and Benefits Compared to Antibiotics. ACS Nano.

[7] Matsuo K., Yoshihara K., Nagaoka N., Makita Y., Obika Y.H., Okihara T., Matsukawa A., Yoshida Y., Van Meerbeek B. (2019). Rechargeable anti-microbial adhesive formulation containing cetylpyridinium chloride montmorillonite. Acta Biomater..

[8] Rawson T.M., Wilson R.C., O’Hare D., Herrero P., Kambugu A., Lamorde M., Ellington M., Georgiou P., Cass A., Hope W.W. (2021). Optimizing antimicrobial use: Challenges, advances and opportunities. Nat. Rev. Microbiol..

[9] Mohd Farid D.A., Zahari N.A.H., Said Z., Ghazali M.I.M., Hao-Ern L., Mohamad Zol S., Aldhuwayhi S., Alauddin M.S. (2022). Modification of Polymer Based Dentures on Biological Properties: Current Update, Status, and Findings. Int. J. Mol. Sci..

[10] Pavanello L., Cortês I.T., de Carvalho R.D.P., Picolo M.Z.D., Cavalli V., Silva L.T.S., Boaro L.C.C., Prokopovich P., Cogo-Müller K. (2024). Physicochemical and biological properties of dental materials and formulations with silica nanoparticles: A narrative review. Dent. Mater..

[11] Adnadjevic B., Jovanovic J. (2009). A comparative kinetics study of isothermal drug release from poly(acrylic acid) and poly(acrylic-co-methacrylic acid) hydrogels. Colloids Surf. B Biointerfaces.

[12] Naoe T., Hasebe A., Horiuchi R., Makita Y., Okazaki Y., Yasuda K., Matsuo K., Yoshida Y., Tsuga K., Abe Y. (2020). Development of tissue conditioner containing cetylpyridinium chloride montmorillonite as new antimicrobial agent: Pilot study on antimicrobial activity and biocompatibility. J. Prosthodont. Res..

[13] Fathilah A.R., Himratul-Aznita W.H., Fatheen A.R., Suriani K.R. (2012). The antifungal properties of chlorhexidine digluconate and cetylpyrinidinium chloride on oral Candida. J. Dent..

[14] Mohapatra S., Mohandas R., Rajpurohit L., Patil S. (2025). Comparative Evaluation of the Efficacy of Cetylpyridinium Chloride Mouthwash and Chlorhexidine Mouthwash in Plaque Reduction: A Systematic Review and Meta-analysis. Curr. Oral Health Rep..

[15] Mao X., Auer D.L., Buchalla W., Hiller K.A., Maisch T., Hellwig E., Al-Ahmad A., Cieplik F. (2020). Cetylpyridinium Chloride: Mechanism of Action, Antimicrobial Efficacy in Biofilms, and Potential Risks of Resistance. Antimicrob. Agents Chemother..

[16] Freire M.C.L.C., Alexandrino F., Marcelino H.R., Picciani P.H.S., Silva K.G.H.E., Genre J., Oliveira A.G., Egito E.S.T.D. (2017). Understanding Drug Release Data through Thermodynamic Analysis. Materials.

[17] Kozaki R., Sato H., Fujishima A., Sato S., Ohashi H. (1996). Activation Energy for Diffusion of Cesium in Compacted Sodium Montmorillonite. J. Nucl. Sci. Technol..

[18] Langa G.P.J., Muniz F.W.M.G., Costa R.d.S.A., da Silveira T.M., Rösing C.K. (2021). The effect of cetylpyridinium chloride mouthrinse as adjunct to toothbrushing compared to placebo on interproximal plaque and gingival inflammation—A systematic review with meta-analyses. Clin. Oral Investig..

[19] Subramanian N., Nielsen Lammers L. (2022). Thermodynamics of ion exchange coupled with swelling reactions in hydrated clay minerals. J. Colloid Interface Sci..

[20] Vila T., Sultan A.S., Montelongo-Jauregui D., Jabra-Rizk M.A. (2020). Oral Candidiasis: A Disease of Opportunity. J. Fungi.

[21] Brown G.D., Ballou E.R., Bates S., Bignell E.M., Borman A.M., Brand A.C., Brown A.J.P., Coelho C., Cook P.C., Farrer R.A. (2024). The pathobiology of human fungal infections. Nat. Rev. Microbiol..

[22] Holmes C.L., Albin O.R., Mobley H.L.T., Bachman M.A. (2025). Bloodstream infections: Mechanisms of pathogenesis and opportunities for intervention. Nat. Rev. Microbiol..

[23] Zaongo S.D., Ouyang J., Isnard S., Zhou X., Harypursat V., Cui H., Routy J.P., Chen Y. (2023). Candida albicans can foster gut dysbiosis and systemic inflammation during HIV infection. Gut Microbes.

[24] Kashyap B., Padala S.R., Kaur G., Kullaa A. (2024). Candida albicans Induces Oral Microbial Dysbiosis and Promotes Oral Diseases. Microorganisms.

[25] Ferro A.C., Spavieri J.H.P., Ribas B.R., Scabelo L., Jorge J.H. (2023). Do denture cleansers influence the surface roughness and adhesion and biofilm formation of Candida albicans on acrylic resin? Systematic review and meta-analysis. J. Prosthodont. Res..

[26] Mylonas P., Milward P., McAndrew R. (2022). Denture cleanliness and hygiene: An overview. Br. Dent. J..

[27] Okeke C.A.V., Khanna R., Ehrlich A. (2023). Quaternary Ammonium Compounds and Contact Dermatitis: A Review and Considerations During the COVID-19 Pandemic. Clin. Cosmet. Investig. Dermatol..

